# Dysmenorrhea and Associated Factors among Polish Women: A Cross-Sectional Study

**DOI:** 10.1155/2020/6161536

**Published:** 2020-07-11

**Authors:** Zofia Barcikowska, Karolina Wójcik-Bilkiewicz, Agnieszka Sobierajska-Rek, Magdalena Emilia Grzybowska, Piotr Wąż, Katarzyna Zorena

**Affiliations:** ^1^Department of Immunobiology and Environment Microbiology, Medical University of Gdańsk, Dębinki 7, 80-211, Gdańsk, Poland; ^2^Department of Clinical Anatomy, Medical University of Gdańsk, Dębinki 1, 80-211, Gdańsk, Poland; ^3^Department of Rehabilitation Medicine, Medical University of Gdańsk, Al. Zwycięstwa 30, 80-219, Gdańsk, Poland; ^4^Department of Gynecology, Gynecological Oncology and Gynecological Endocrinology, Medical University of Gdańsk, Smoluchowskiego 17, 80-214, Gdańsk, Poland; ^5^Department of Nuclear Medicine, Medical University of Gdańsk, Tuwima 15, 80-210, Gdańsk, Poland

## Abstract

**Purpose:**

The aim of the research was to conduct an assessment of the prevalence of dysmenorrhea and associated factors among Polish women. *Patients and Methods*. A cross-sectional study was conducted among Polish women using an online questionnaire. The mean age of the participants was 23 ± 4 years. Out of the total of 1,317 women who took part in the study, 1,127 were included in the analysis, and 190 were excluded due to incomplete answers. The questionnaire consisted of 19 questions that were grouped into three parts. The first concerned sociodemographic data such as age, weight, education, and residence (urban or rural). The second part of the questionnaire pertained to the factors of dysmenorrhea (premenstrual syndrome, age of menarche, and family history of dysmenorrhea.). In the third part, the women were asked about their diet, alcohol intake, cigarette smoking, and physical activity.

**Results:**

Dysmenorrhea affected 94% of the interviewed women. Dysmenorrhea was most likely to occur among respondents whose mothers had a history of dysmenorrhea (*p* < 0.005). Significant relationship between the occurrence of dysmenorrhea among respondents and their sisters was also observed (*p* < 0.005). The prevalence of premenstrual syndrome (PMS) was significantly higher in women reporting dysmenorrhea (*p* < 0.005). Other significant factors associated with dysmenorrhea were age of menarche (*p* < 0.005), stress frequency (*p*=0.005), lack of physical activity (*p*=0.037), and self-esteem (*p*=0.042). However, in the respondents, no significant relationship was observed between dysmenorrhea and diet, smoking, body mass index, and alcohol intake.

**Conclusion:**

The study points to the fact that the problem of dysmenorrhea affects many Polish women. Women with dysmenorrhea were characterized with a family history of dysmenorrhea, occurrence of PMS, early age of menarche, stressful lifestyle, lack of physical activity, and low self-esteem. We suggest that further assessment of factors contributing for dysmenorrhea among women is necessary.

## 1. Introduction

Dysmenorrhea, also known as painful periods, is a syndrome resulting in painful cramps during menstruation [1]. Dysmenorrhea often occurs among young women and often compounds their quality of life. A great majority of women are forced to miss full days off school or work, due to dysmenorrhea, and have difficulty concentrating on daily activities [1–3]. The women with dysmenorrhea often report cancelling social meetings, have difficulties falling asleep, or feel depressed and irritated during menstruation [2, 3]. According to the World Health Organization (WHO) data, dysmenorrhea affects between 1.7% and 97% of women [4]. In the two existing Polish epidemiological studies, it was demonstrated that dysmenorrhea is experienced by 65% of Polish women [5, 6].

Dysmenorrhea is classified as primary and secondary [2]. Primary dysmenorrhea is attributable to excessive, pathological uterine contractions, without any other pathologic changes in the area of the pelvis minor [2]. Most commonly it appears earlier than 12 months after menarche. The pain often occurs during the first 8–72 hours [2]. Secondary, dysmenorrhea is associated with a prevalence of acquired changes, such as endometriosis, chronic inflammatory condition of the pelvis minor, uterine fibroids, endometrial polyps, and cervix stenosis, as well anatomical and functional abnormalities of the generative organs [1, 3]. Usually, dysmenorrhea affects women aged 20–25, often easing off after the first pregnancy [7, 8]. During the menstrual cycle, discomfort starts a few hours before or directly with the menses. The pain is characterized as systolic, often radiating towards the groin, back, or thighs [3, 9]. Abdominal pain may be accompanied by other symptoms, such as nausea, headache, back pain, diarrhoea, or fatigue [3, 10].

Despite many studies on dysmenorrhea, the etiopathogenesis of this condition has not been fully investigated. However, it is known that susceptibility to dysmenorrhea may be associated with genetic factors [11–13]. It is also known that the occurrence of dysmenorrhea in women is associated with an elevated secretion of prostaglandins and other inflammatory factors [3, 14, 15]. Prostaglandins stimulate excessive contractile function of the uterus, that leads to the reduction of blood flow and hypoxia of this organ. Excessive release of prostaglandins also explains the coexistence of other symptoms, such as nausea and headache [10, 16, 17].

In addition, there are more scientific reports about factors that may predispose women to the occurrence of pain during menstruation, including body mass index (BMI), socioeconomic situation, dietary habits, and stress levels, although the research results are contradictory [6, 18, 19]. Therefore, the purpose of this cross-sectional study was to assess the prevalence of dysmenorrhea and related factors among Polish women.

## 2. Materials and Methods

### 2.1. Design and Data Collection

This was a cross-sectional study conducted among Polish women using an online questionnaire. A web-based survey available through Google forms was completed by 1,317 respondents. The information about the study was spread by social media, and the questionnaire was available as open access. An informed consent was obtained in the first page of the study's questionnaire. It explained the aims of the study, emphasized the confidentiality of the filled out information, and provided information about the affiliation of the researchers. Participants were able to withdraw from the study at any point. No identifying information was obtained through the questionnaire, and all collected data were solely used for the statistical analysis. The questionnaire was prepared with reference to previous studies in the literature [20–23]. To maximize comprehension of the questionnaire, it was piloted in a sample of 10 women aged 20–35. The questionnaire was designed in Polish for the purpose of this study, and then translated into English for the purpose of publication. It consisted of 19 questions (Appendix A). Multiple choice, single-answer questions, and open questions were included. The questions were grouped into three parts. The first concerned sociodemographic data, such as age, weight, education, and residence (urban or rural). The second part of the questionnaire pertained to the factors of dysmenorrhea. The respondents were asked about the regularity of their menstrual cycles, dysmenorrhea, its intensity and duration (based on the average of all past menstrual cycles), premenstrual syndrome (PMS), menarche age, and the family history of dysmenorrhea. In the third part, the women were asked about their diet, alcohol intake, cigarette smoking, and physical activity. They were also asked to assess how often they experienced stress and how they perceived themselves: whether they were satisfied with their appearance and whether they accepted themselves or not.

The respondents' BMI was calculated based on the answers provided to questions about their height and weight. The results were divided into 3 groups based on the WHO classification: underweight (<18.5), normal weight (18.5–24.9), and overweight (25<) [24].

Pain severity was assessed with the use of the Numeric Pain Rating Scale (NPRS) [25]. The NPRS is an 11 point scale from 0 to 10, where 0 indicates “no pain”, and 10 indicates the “worst imaginable pain”. Respondents were requested to choose a single number from the scale, that best indicates their level of pain. Only the subjects declaring dysmenorrhea assessed the intensity of pain. The analysis did not include the “0” points answer. Based on the literature, the pain was classified as mild, moderate, or severe. Mild pain was indicated by 1–3 points, moderate by 4–6 points, and severe by 7–10 points [26]. For the purpose of the analysis, the women were divided into three groups: women with dysmenorrhea during every cycle (PG), women with dysmenorrhea occasionally only (SG), and women without dysmenorrhea (WG).

### 2.2. Data Analysis

The results were generated using the *R* statistics language [27]. Basic statistics (mean, standard deviation, and median) were calculated for quantitative variables. The Shapiro–Wilk test was used to test for normality. The differences between the values of more than two data sets were analysed using the Kruskal–Wallis test and the post hoc Wilcoxon rank sum test. Qualitative variables were characterized by counts and percentages. Pearson's chi-squared test and Fisher's exact test for counting data were used to test the independence of these variables. The strength of the relationship between events (described by qualitative variables) was determined by calculating the odds ratios (OR) and their confidence intervals (CIs). The values given in the tables for the qualitative variables were also used to create the association diagrams [28]. The figures show, among others, the Pearson residuals and *p* values for the independence test. In the association diagrams, the blue colour indicates numbers that are higher than the expected. The red colour shows cases where the numbers are smaller than the expected. In contrast, the grey colour represents the data where the numbers are close to the expected, i.e., the null hypothesis of the independence test is true. In the association chart, each cell of the contingency table is represented by a rectangle. In our work, the rectangle has a height proportional to the Pearson residual and a width proportional to the root of the expected value, thanks to which the surface area is equal to the difference between observed and expected frequencies. The rectangles in each row are set in relation to the baseline indicating independence. The correspondence analysis [29] was used to search for clusters of variables creating the tables with more than two rows and columns. The assumed significance level is *α* = 0.05.

## 3. Results

### 3.1. Sociodemographic Factors among Polish Respondents

Out of the total of 1,317 women who took part in the study, 1,127 were included in the analysis, and 190 were excluded due to incompleteness of the answers. The response rate was 93.2%. The mean age of the participants was 23 ± 4 years. It was observed that among 1,127 respondents, 928 (82.3%) had regular and 199 (17.7%) irregular of menstrual cycle. The majority of the respondents were university students (54.8%). Over 96% of the respondents lived in urban areas. Among 1,127 respondents, 1,059 (94%) had dysmenorrhea, including 610 (54.1%) every menstrual cycle and 449 (39.9) not every menstrual cycle. However, only 68 (6.0%) did not experience dysmenorrhea at all (Table 1).

### 3.2. Pain Severity among Women with Dysmenorrhea

Based on the NPRS scale, the study showed that 68 (6.0%) of the respondents did not experience dysmenorrhea, while 797 (70.7%) with dysmenorrhea rated the pain as severe. Moderate pain affected 233 (20.7%) of women, and only 29 (2.6%) women evaluated their pain as mild (Table 2).

### 3.3. Analysis of Factors Associated with Dysmenorrhea among Respondents

Factors associated with dysmenorrhea among respondents are provide in Table 3

#### 3.3.1. Dysmenorrhea in Mothers of the Respondents

**Figure 1:**
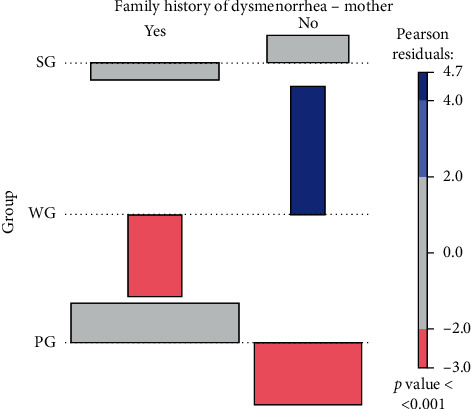
A diagram of multidimensional data association for respondents from the groups (PG, SG, and WG) and the occurrence of dysmenorrhea in the respondents' mothers (answers: yes, no). A significant statistical relationship *p* < 0.001 was found between the multidimensional data. PG, women with dysmenorrhea during every cycle, SG, women with dysmenorrhea occasionally, and WG, women without dysmenorrhea.

Pearson's chi-squared test showed that dysmenorrhea was most likely to occur in respondents whose mothers had a history of dysmenorrhea (*p* < 0.005) (Table 3). The odds ratio (OR) for the occurrence of dysmenorrhea in mothers of the women from the PG group in relation to mothers of the women from the WG group was 6.23 (3.33–11.64 CI). Model methods were used to analyse nonmetric variables, such as total distribution. The analysis, conducted using contingency tables, made it possible to assess the relationship between multidimensional data for the respondents from individual groups (PG, SG, and WG) and the occurrence of dysmenorrhea in the respondents' mothers (answers: yes/no). The analysis showed that the dysmenorrhea in mothers of the women from the WG group was less frequent than the expected value (red). However, the absence of pain in mothers of the women from the WG group was more frequent than the expected value (blue). The occurrence of dysmenorrhea in mothers of the women from the SG and PG groups as well as the absence of dysmenorrhea in mothers of the women from the SG group were in line with the expected values. The analysis of multidimensional data, conducted using contingency tables, demonstrated a statistically significant relationship *p* < 0.001 (Figure 1).

#### 3.3.2. Dysmenorrhea in Sisters of the Respondents

Pearson's chi-squared test showed that dysmenorrhea was most likely to occur in respondents whose sisters had a history of dysmenorrhea (*p* < 0.005) (Table 3). The OR for the occurrence of dysmenorrhea in sisters of the women from the PG group in relation to sisters of the respondents from the WG group was 3.17 (1.48–6.80 CI). In order to measure the relationship between the multivariate variables, the analysis using contingency tables was conducted for the respondents from individual groups (PG, SG, and WG) and the occurrence of dysmenorrhea in sisters of the respondents (answers: yes/ no).

**Figure 2:**
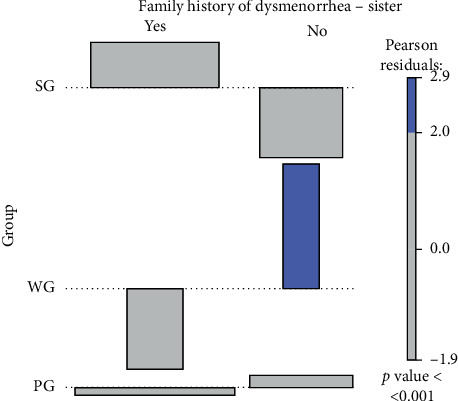
A diagram of multidimensional data association for respondents from individual groups (PG, SG, and WG) and the occurrence of dysmenorrhea in sisters of the respondents (answers: yes, no). A significant statistical relationship *p* < 0.001 was found between the multidimensional data. PG, women with dysmenorrhea during every cycle, SG, women with dysmenorrhea occasionally, and WG, women without dysmenorrhea.

Dysmenorrhea occurred less often than expected in sisters of the women from the WG group (blue). The number of sisters of the women from the PG and SG groups who had dysmenorrhea as well as those who did not suffer from dysmenorrhea was close to the expected value (grey). The occurrence of dysmenorrhea in sisters of the respondents from the WG group was also close to the expected value. The multivariate analysis, conducted using contingency tables for the study parameters, showed a statistically significant relationship *p* < 0.001 (Figure 2).

#### 3.3.3. Premenstrual Syndrome (PMS)

**Figure 3:**
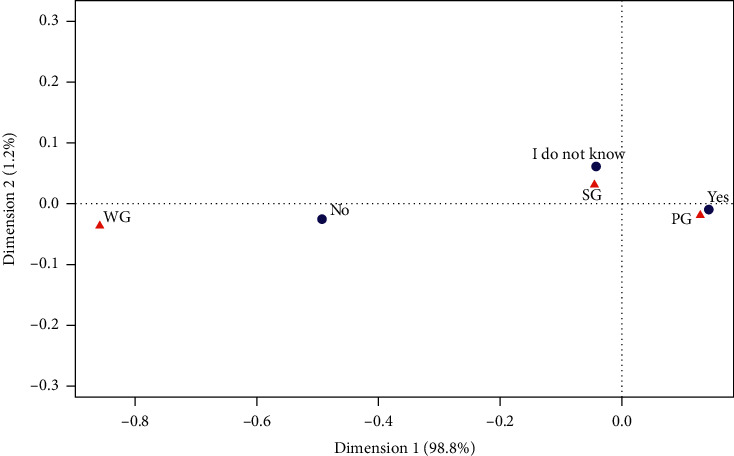
The analysis of correspondence regarding premenstrual syndrome and dysmenorrhea among the studied female respondents. PG, women with dysmenorrhea during every cycle, SG, women with dysmenorrhea occasionally, and WG, women without dysmenorrhea.

The correspondence analysis was conducted to determine the relationship between responses of the respondents to the questions regarding the perception of dysmenorrhea and the occurrence of PMS. It has been shown that there is a strong relationship between the responses: “yes” to the occurrence of dysmenorrhea and “yes” to suffering from PMS. A strong relationship was also found between the “I do not know” answer to the occurrence of PMS and the answer “sometimes” in the question about dysmenorrhea (Figure 3). The OR for PMS in the women from the PG group in relation to the WG group was 2.27 (1.1–4.5 CI). Pearson's chi-squared test showed a statistically significant relationship between the perception of pain and the occurrence of PMS (*p* < 0.005) (Table 3).

#### 3.3.4. Self-Esteem

**Figure 4:**
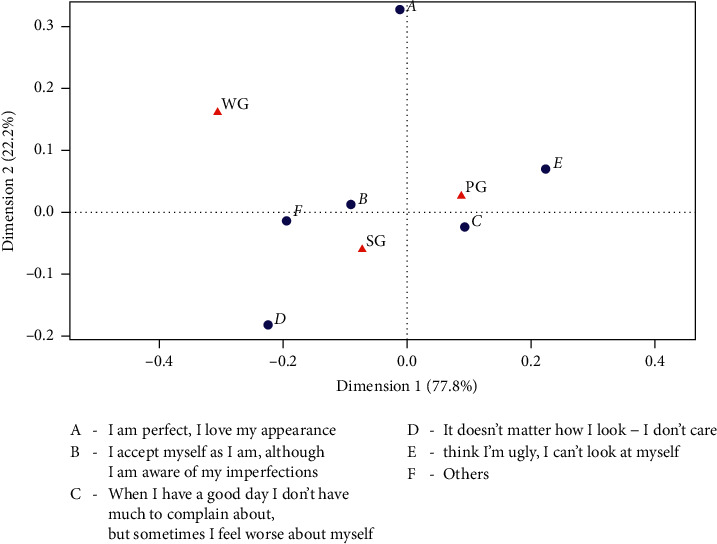
The analysis of correspondence regarding self-esteem and dysmenorrhea among the studied female respondents. The questions are included in Appendix A. PG, women with dysmenorrhea during every cycle, SG women with dysmenorrhea occasionally, and WG, women without dysmenorrhea.

The correspondence analysis performed for self-esteem vs. dysmenorrhea showed a relationship between the answer “I think I am ugly, I cannot look at myself” and the occurrence of dysmenorrhea (Figure 4). Fisher's exact test detected that the relationship is statistically significant (*p*=0.042) (Table 3) (All the questions are included in Appendix A).

#### 3.3.5. Age of Menarche

**Figure 5:**
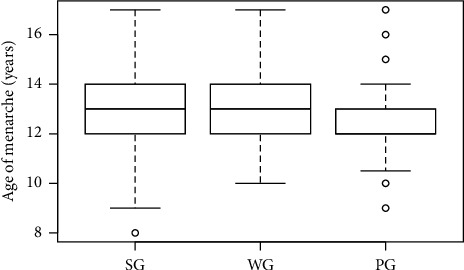
Age of the first menstruation and dysmenorrhea in respondents. A statistically significant difference was revealed between the PG and SG groups (*p*=0.011), as well as between the PG and WG (*p*=0.002), and between the WG and SG (*p*=0.045). PG, women with dysmenorrhea during every cycle, SG, women with dysmenorrhea occasionally, and WG, women without dysmenorrhea.

In our study, 542 (48%) women had their first menstruation at ≤12. The age of menarche in each of the analysed groups was not normally distributed. The Kruskal–Wallis test showed the relation between the age of first menstruation and the prevalence of dysmenorrhea (*p* < 0.005) (Table 3). Post hoc tests for the Kruskal–Wallis analysis revealed statistically significant differences between all groups: *p*=0.011 for PG–SG, *p*=0.002 for PG–WG, and *p*=0.045 for WG–SG (Figure 5).

#### 3.3.6. Frequency of Stress

It was shown that 164 (26.9%) women from the PG group experienced stress every day, 273 (44.7%) several times a week, 134 (22.0%) several times a months, and only 39 (6.4%) of those questioned in this group claimed to rarely experience stress. Similar results were obtained in the SG group: 100 (22.3%), 177 (39.4%), 124 (27.6%), and 48 (10.7%), respectively. In the WG group, it was observed that 13 (19.1%) of respondents experienced stress every day, 28 (41.2%) several times a week, 16 (23.5%) several times a month, and 11 (16.2%) rarely. The result of the independence test for the examined groups of variables is statistically significant (*p*=0.005) (Table 3).

#### 3.3.7. Diet, Physical Activity, Alcohol Intake, Cigarette Smoking, and BMI

Women who reported no physical activity, significantly more often experienced dysmenorrhea (*p* = 0.037). However, the frequency of physical exercise did not significantly affect dysmenorrhea (*p*=0.564). The women were asked “How often do you exercise?” and could choose an answer from the following: “every day,” “several times a week,” “once a week,” “once a month,” “seasonally,” “rarely,” and “never” (Table 3).

The respondents answered the question whether their diet was healthy. There were three answers to choose from: “yes,” “no,” and “I do not know.” In the studied group of respondents, there was no statistical significance between diet and dysmenorrhea (*p*=0.068), as well as between BMI and dysmenorrhea (*p*=0.271). Based on the respondents' answers, it was concluded that cigarette smoking did not affect the occurrence of dysmenorrhea (*p*=0.924). Moreover, the analysis showed that the frequency of alcohol intake did not affect the frequency of dysmenorrhea (*p*=0.396). The answer options were the following: “I drink alcohol every day”, “I drink alcohol several times a month,”.“I drink alcohol at parties,” “I rarely drink alcohol,” and “I never drink alcohol” (Table 3).

## 4. Discussion

In the studied group of respondents, no statistically significant relationship was found between diet, BMI, frequency of alcohol intake, cigarette smoking, and dysmenorrhea in Polish women. Our results confirm previous reports of other researchers, that amount of alcohol intake had no significant effect on the occurrence of dysmenorrhea [30]. A healthy or unhealthy diet also does not affect the frequency of dysmenorrhea in the studied women. However, according to the literature, an unhealthy diet could predispose women to the occurrence of dysmenorrhea [31, 32].

In our studied group, there was no relationship between BMI and dysmenorrhea. This might be due to the fact that the majority of women who participated in the research presented BMI values from 18.5 to 24.9 kg/m [2]. Those values are regarded as normal weight [24]. There were few participants, in the research, whose BMI would indicate being either underweight or overweight. Our results are consistent with the research by Hailemeskel et. al. [19], where the majority of respondents' BMI values were normal [19]. In contrast, several studies have shown that an abnormal BMI could predispose women to dysmenorrhea [5, 33]. The researchers observed that obese women who have a significant accumulation of visceral fat tissue, most frequently struggle with painful menstrual periods [6].

Our study found that dysmenorrhea affects 94% of respondents. We have observed that some factors appear more frequently in women with dysmenorrhea. The statistically significant factor was the occurrence of dysmenorrhea in the family—in mothers and sisters. The respondents in the group PG confirmed that their mothers and sisters often suffered from dysmenorrhea. Pearson's chi-squared test and the multidimensional data analysis, conducted using contingency tables, showed a statistically significant relationship between the occurrence of dysmenorrhea in the mothers and sisters of the respondents. Our result is supported by other studies [20, 34]. Polat et al. showed that the daughters of women who suffered from menstrual complaints were also affected by menstrual discomfort [34]. The authors suggest that the prevalence of dysmenorrhea among daughters could be associated with behaviours learnt from mothers [20].

Premenstrual syndrome (PMS) is a syndrome present during the luteal phase of the menstrual cycle and subsides at the time of menstrual bleeding, and it is characterized by psychological and physical symptoms, such as breast hypersensitivity, oedema, irritability, and depressed mood [35–39]. Our research demonstrates that the prevalence of PMS was significantly higher in the PG group. Premenstrual syndrome was detected in 83.8% of women with dysmenorrhea and only in 39.3% of women who had not reported dysmenorrhea. A similar conclusion that PMS often accompanies dysmenorrhea was reported by Kitamura et al. [40]. A correlation between PMS and dysmenorrhea was observed in girls aged 15–19 [40].

Our study also showed that the age of the first menstruation had a significant relationship to the occurrence of dysmenorrhea. As many as 48% of the respondents had their first menstruation at the age of ≤12. Our study results are consistent with the findings of other authors who demonstrated that the occurrence of dysmenorrhea increases with a lower age of the first menstruation [8, 18, 23]. On the other hand, there are studies showing no relationship between the lower age of the first menstrual period and dysmenorrhea [20].

Self-esteem was another statistically significant factor analysed in our research. We have shown a significant relationship between the answer “I think I am ugly, I cannot look at myself” and the occurrence of dysmenorrhea. To our knowledge, this is the first research into self-esteem in the context of dysmenorrhea. The significance of our results indicates a need for further research into the impact of self-esteem on the occurrence of dysmenorrhea.

Our results confirm that the physical activity has an impact on the occurrence of dysmenorrhea. Various researchers presented their own training programs to reduce dysmenorrhea in women [39, 40]. The set of exercises proposed by Kitamuraet al. [40] reduced the severity of dysmenorrhea, but also had an influence on the duration and amount of blood loss during menstruation. In addition, Kannan et. al. [41] demonstrated that the physical activity has an impact on the concentrations of progesterone, prostaglandin, and the tumour necrosis factor.

Our study showed that stress significantly contributes to dysmenorrhea. Almost 30% of women who suffered from dysmenorrhea during every cycle claimed to have been experiencing stress every day, and only 6.8% of women in this group rarely suffered from pain. In the group of women without dysmenorrhea, 20% experienced stress and almost 17% were rarely stressed. For comparison, in a study performed in India, stress affected only 24.4% of women with dysmenorrhea [42]. In our study group, 54.8% were students. Previous studies have indicated that the life of Polish students is stressful [43]. In Poland, within the last 20 years, the number of university students has increased more than 400%. Moreover, since the political and economic transformation in Poland, more and more students combine studying with work. It was shown that Polish students are at the top of the European ranking concerning the hardest-working young people who study and work simultaneously [44].

## 5. Study Limitations

The present study has some limitations that we would like to address. First, due to the questionnaire's distribution method, the sample cannot be treated as a representative for the whole population of Polish women. The study mostly reflects the condition of young (20–25) women from urban areas. Second, we cannot verify the responses of women with dysmenorrhea. Further cohort and longitudinal studies are being conducted to verify our findings and to garner a deeper understanding.

## 6. Conclusion

The study points to the fact that the problem of dysmenorrhea affects many Polish women. Women with dysmenorrhea were characterized with a family history of dysmenorrhea, occurrence of PMS, early age of menarche, stressful lifestyle, lack of physical activity, and low self-esteem. Therefore, we suggest that assessment of factors that contribute to dysmenorrhea is needed. Understanding the aetiology and factors responsible for dysmenorrhea will allow a precise diagnosis to be made and thus the application of more effective therapeutic methods.

## Figures and Tables

**Table 1:** Sociodemographic characteristics of women participants in the study group.

Variable	*n* (%)	
Age (mean, SD)	23 ± 4	1127 (100%)
Education	Pupil	25 (2.2)
	University student	618 (54.8)
	Primary	9 (0.8)
	Secondary	204 (18.1)
	Higher	271 (24.1)
Place of residence	Rural	39 (3.5)
	Urban	1088 (96.5)
Regularity of cycle	Irregular	199 (17.7)
	Regular	928 (82.3)
Dysmenorrhea occurrence	Yes, every menstrual cycle	610 (54.1)
	Yes, not every menstrual cycle	449 (39.9)
	No	68 (6.0)

Abbreviations: SD, standard deviation.

**Table 2:** Pain intensity in NPRS (among women with dysmenorrhea).

	*n* (%)
No pain (0)	68 (6.0)
Mild (1–3)	29 (2.6)
Moderate (4–6)	233 (20.7)
Severe (7–10)	797 (70.7)

Abbreviations: NPRS, numeric pain rating scale.

**Table 3:** Factors associated with dysmenorrhea among respondents.

		PG	SG	WG	*p* value
		*n* (%)	*n* (%)	*n* (%)	
Family history of dysmenorrhea, mother	Yes	364 (59.7)	188 (41.9)	17 (25.0)	<0.005^*∗*^
	No	110 (18.0)	89 (19.8)	32 (47.1)	
	I do not know	136 (22.3)	172 (38.3)	19 (27.9)	

Family history of dysmenorrhea, sister	Yes	174 (28.5)	125 (27.8)	13 (19.1)	<0.005^*∗*^
	No	76 (12.5)	36 (8.0)	18 (26.5)	
	I do not know	46 (7.5)	59 (13.2)	7 (10.3)	
	I do not have a sister	314 (51.5)	229 (51.0)	30 (44.1)	

PMS	Yes	421 (69.0)	273 (60.8)	21 (30.9)	<0.005^*∗*^
	No	78 (12.8)	83 (18.5)	33 (48.5)	
	I do not know	111 (18.2)	93 (20.7)	14 (20.6)	

Age of menarche	≤12 years	316 (51.8)	205 (45.7)	21 (30.9)	<0.005^*∗*^
	13-14 years	248 (40.7)	193 (43.0)	36 (52.9)	
	≥15 years	46 (7.5)	51 (11.3)	11 (16.2)	

Healthy diet	Yes	259 (42.5)	215 (47.9)	36 (52.9)	0.068
	No	218 (35.7)	154 (34.3)	15 (22.1)	
	I do not know	133 (21.8)	80 (17.8)	17 (25.0)	

Stress	Everyday	164 (26.9)	100 (22.3)	13 (19.1)	0.005^*∗*^
	A few times a week	273 (44.7)	177 (39.4)	28 (41.2)	
	A few times a month	134 (22.0)	124 (27.6)	16 (23.5)	
	Rarely	39 (6.4)	48 (10.7)	11 (16.2)	

Self-esteem	I am perfect, I love my appearance	16 (2.6)	7 (1.6)	3 (4.4)	0.042^*∗*^
	I accept myself as I am, although I am aware of my imperfections	271 (44.4)	225 (50.1)	41 (60.3)	
	When I have a good day, I do not have much to complain about, but sometimes I feel worse about myself	262 (42.9)	175 (39.0)	18 (26.5)	
	It does not matter how I look—I don't care	6 (1.0)	8 (1.8)	1 (1.5)	
	I think I am ugly, I cannot look at myself	40 (6.6)	19 (4.2)	2 (2.9)	
	Other	15 (2.5)	15 (3.3)	3 (4.4)	

Exercise	Yes	311 (51.0)	260 (57.9)	42 (61.8)	0.037^*∗*^
	No	299 (49.0)	189 (42.1)	26 (38.2)	

Frequency of exercises	Everyday	21 (3.4)	15 (3.3)	3 (4.4)	0.5636
	A few times a week	196 (32.0)	165 (36.7)	26 (38.2)	
	Once a week	84 (13.8)	71 (15.8)	10 (14.7)	
	Once a month	14 (2.3)	9 (2.0)	1 (1.5)	
	Seasonally	84 (13.8)	51 (11.4)	12 (17.7)	
	Rarely	94 (15.4)	51 (11.4)	6 (8.8)	
	Never	118 (19.3)	87 (19.4)	10 (14.7)	

BMI	<18.5	59 (9.7)	35 (7.8)	6 (8.8)	0.271
	18.5–24.9	474 (77.7)	341 (75.9)	52 (76.5)	
	>25	77 (12.6)	73 (16.3)	10 (14.7)	

Alcohol intake	Everyday	2 (0.3)	2 (0.5)	0 (0.0)	0.3602
	A few times a week	53 (8.7)	34 (7.6)	4 (5.9)	
	Several times a month	177 (29.0)	156 (34.7)	29 (42.7)	
	During celebrations, parties	263 (43.1)	177 (39.4)	22 (32.3)	
	Never	34 (5.6)	16 (3.6)	3 (4.4)	
	Very rarely	81 (13.3)	64 (14.2)	10 (14.7)	

Smoking	Yes	123 (20.2)	95 (21.2)	14 (20.6)	0.925
	No	487 (79.8)	354 (78.8)	54 (79.8)	

Abbreviations: PG, women with dysmenorrhea during every cycle, SG, women with dysmenorrhea occasionally, WG, women without dysmenorrhea, PMS, premenstrual syndrome, and BMI, body mass index. ^*∗*^Statistically significant variables.

## Data Availability

The data used to support the findings of this study are included within the article.

## Appendix

Questionnaire–English version

(1) Weight (kg)
(2) Height (m)
(3) Age (years)
(4) Place of origin
  - Rural
  - Urban
(5) Education
  - School student
  - Student
  - Primary education
  - Secondary education
  - Higher education
(6) Age of menarche (your first menstruation)?
(7) Are your menstrual cycles regular (21–35 days)?
  - Yes
  - No
(8) Do you suffer from menstrual pain (dysmenorrhea)?
  - Yes, dysmenorrhea during every or from time to time menstruation
  - No, without dysmenorrhea
(9) Has your mother suffered from menstrual pain?
  - Yes
  - No
  - I do not know
(10) Has your sister (s) suffered from menstrual pain?
  - Yes
  - No
  - I do not know
  - I do not have any sisters
(11) On a scale (NPRS) from 0 to 10, how strongly do you feel menstrual pain at the worst moment of the cycle?
(12) Do you suffer from premenstrual syndrome (PMS)?
  - Yes
  - No
  - I do not know
(13) Do you do some sports?
  - Yes
  - No
(14) How often do you exercise?
  - Every day
  - A few times a week
  - Once a week
  - Once a month
  - Seasonally
  - Very rarely
  - I do not exercise
(15) Do you think, that your diet is healthy?
  - Yes
  - No
  - I do not know
(16) Do you smoke?
  - Yes
  - No
(17) How often do you drink alcohol?
  - Everyday
  - A few times a week
  - Several times a month
  - During celebrations, parties
  - Very rarely
  - Never
(18) How do you perceive yourself?
  - I am perfect. I love my appearance
  - I accept myself as I am, although I am aware of my imperfections
  - When I have a good day, I do not have much to complain about, but sometimes I feel worse about myself
  - It does not matter how I look—I do not care
  - I think I am ugly, I cannot look at myself
  - Others
(19) How often to you experience stress?
  - Every day
  - A few times a week
  - Several times a month
  - I experience stress rarely
